# IQGAP1 is a key node within the small GTPase network

**DOI:** 10.4161/sgtp.27451

**Published:** 2013-12-19

**Authors:** Guillaume Jacquemet, Martin J Humphries

**Affiliations:** Wellcome Trust Centre for Cell-Matrix Research; Faculty of Life Sciences; University of Manchester; Manchester, UK

**Keywords:** small GTPase crosstalk, IQGAP1, Ras GTPases, small GTPase network, signal transduction

## Abstract

Coordination of the activity of multiple small GTPases is required for the regulation of many physiological processes, including cell migration. There are now several examples of functional interplay between small GTPase pairs, but the mechanisms that control GTPase activity in time and space are only partially understood. Here, we build on the hypothesis that small GTPases are part of a large, integrated network and propose that key proteins within this network integrate multiple signaling events and coordinate multiple small GTPase activities. Specifically, we identify the scaffolding protein IQGAP1 as a master regulator of multiple small GTPases, including Cdc42, Rac1, Rap1, and RhoA. In addition, we demonstrate that IQGAP1 promotes Arf6 activation downstream of β1 integrin engagement. Furthermore, following literature-curated searches and recent mass spectrometric analysis of IQGAP1-binding partners, we report that IQGAP1 recruits other small GTPases, including RhoC, Rac2, M-Ras, RhoQ, Rab10, and Rab5, small GTPase regulators, including Tiam1, RacGAP1, srGAP2 and HERC1, and small GTPase effectors, including PAK6, N-WASP, several sub-units of the Arp2/3 complex and the formin mDia1. Therefore, we propose that IQGAP1 acts as a small GTPase scaffolding platform within the small GTPase network, and recruits and/or regulates small GTPases, small GTPase regulators and effectors to orchestrate cell behavior. Finally, to identify other putative key regulators of small GTPase crosstalk, we have assembled a small GTPase network using protein-protein interaction databases.

The small GTP-binding proteins (GTPases) act as molecular switches to control virtually every cellular process, including cell proliferation (Ras family), membrane trafficking (Arf and Rab families), cytoskeletal dynamics (Rho family) and nuclear transport (Ran family).^1^ Together, these GTPases orchestrate multiple physiological processes, including embryogenesis, polarity, adhesion, migration, and cell fate. Dysregulated GTPase activity has been implicated in a variety of human pathologies including cancer development, progression and dissemination, inflammation, vascular diseases, mental retardation, and infection.^1^^-^^3^

Small GTPases cycle between an active GTP-bound form and an inactive GDP-bound form. The GTPase activation-state dictates function through recruitment of different binding partners (including downstream effectors and GTPase regulators). The cycling of small GTPases between active and inactive states is tightly controlled by GTPase-activating proteins (GAPs) and guanine nucleotide exchange factors (GEFs).^4^^-^^6^ Classically, small GTPases are thought to be activated by transmembrane receptors such as integrins or growth factor receptors, in response to environmental cues.^1^^,^^2^^,^^4^ Accumulating evidence suggests that GTPases can also be regulated by other GTPase family members, share common GTPase regulators, and converge on the same signaling pathway.^7^^-^^10^ This interplay between small GTPases is crucial for the regulation of complex physiological processes, such as directional cell migration, that require the integration of multiple signaling cues at specific times and places.^11^ Indeed, directional cell migration is achieved by spatial and temporal coordination of multiple small GTPases that regulate actin cytoskeleton dynamics, membrane protrusion and the delivery and activation of receptors, including integrins, at new cell-matrix adhesion sites.^12^^,^^13^ It is now well established that the small GTPases Rac1 and RhoA, through opposing roles and antagonistic activities, are required for directional cell migration. The balance between Rac1/RhoA defines actin cytoskeleton structure, cell-matrix adhesion stability and membrane protrusion dynamics and consequently dictates the mode of invasive cell migration.^9^^,^^14^^,^^15^ Other examples of known crosstalk between small GTPases include interplay between the small GTPases Cdc42 and Rac1, Rac1 and RhoG, Rac1 and Arf6,^16^^-^^18^ Arf6 and several Rabs (Rab11, Rab7, Rab8),^19^^-^^21^ and connections between Rab GTPases, Ras, and Rac1.^22^ Recent mass spectrometric analysis of Rac1-binding partners identified over 25 regulators of GTPase activity including specific modulators of Rac1 and several modulators of RhoA, Ras, Rap1 and Arf5, thereby revealing putative novel crosstalk between these GTPases.^23^ Thus, the basic mechanisms of single GTPase regulation within cells are fairly well understood and crosstalk between small GTPase pairs are starting to be elucidated. However, how signaling from multiple GTPases (3 or more) is coordinated to mediate complex processes, such as directional cell migration, remains unknown. It is likely that such coordination is achieved by the existence of specialized protein hubs that integrate and decipher multiple signaling events leading to the spatial and temporal regulation of multiple small GTPase activities.

We and others have identified IQ-motif-containing GTPase-activating protein 1 (IQGAP1), a scaffolding protein involved in the regulation of actin and microtubule organization, cell migration and cell proliferation, as a key regulator of multiple small GTPases and small GTPase functions (Fig. 1).^15^^,^^23^^-^^25^ Supporting a role for IQGAP1 regulating small GTPase functions, Gene Ontology analysis of IQGAP1-binding proteins, identified by mass spectrometry,^23^ revealed an enrichment of proteins involved in “Small GTPase-mediated signal transduction” (Fig. 1). The diverse roles of IQGAP1 may be dictated by IQGAP1 subcellular localization and IQGAP1-binding partners. IQGAP1 accumulates at the cell leading edge during cell migration and at cell-cell junctions, where it is believed to alter junction stability through direct binding to cadherins. IQGAP1 has also been implicated in tumorigenesis as supported by IQGAP1 overexpression in multiple carcinomas and distinct membrane localization in several tumors.^26^^,^^27^ Several proteins (e.g., protein 4.1 and ILK) and signaling events (e.g., integrin activation and PIP2 production) have been demonstrated to regulate IQGAP1 relocalization/specific recruitment within the cell and may be key in initiating IQGAP1 function.^23^^,^^28^^,^^29^ IQGAP1 has been shown to associate directly with and to regulate the activity of multiple small GTPases, both positively and negatively.^24^^,^^25^ However, IQGAP1 does not act directly as either a GEF or a GAP as it contains an inactive RasGAP domain.^30^ Instead, IQGAP1 regulates the activity of small GTPases by either stabilizing the active or inactive GTPase forms and/or through the recruitment of multiple GTPase activity modulators.^15^^,^^23^^,^^25^

**Figure:**
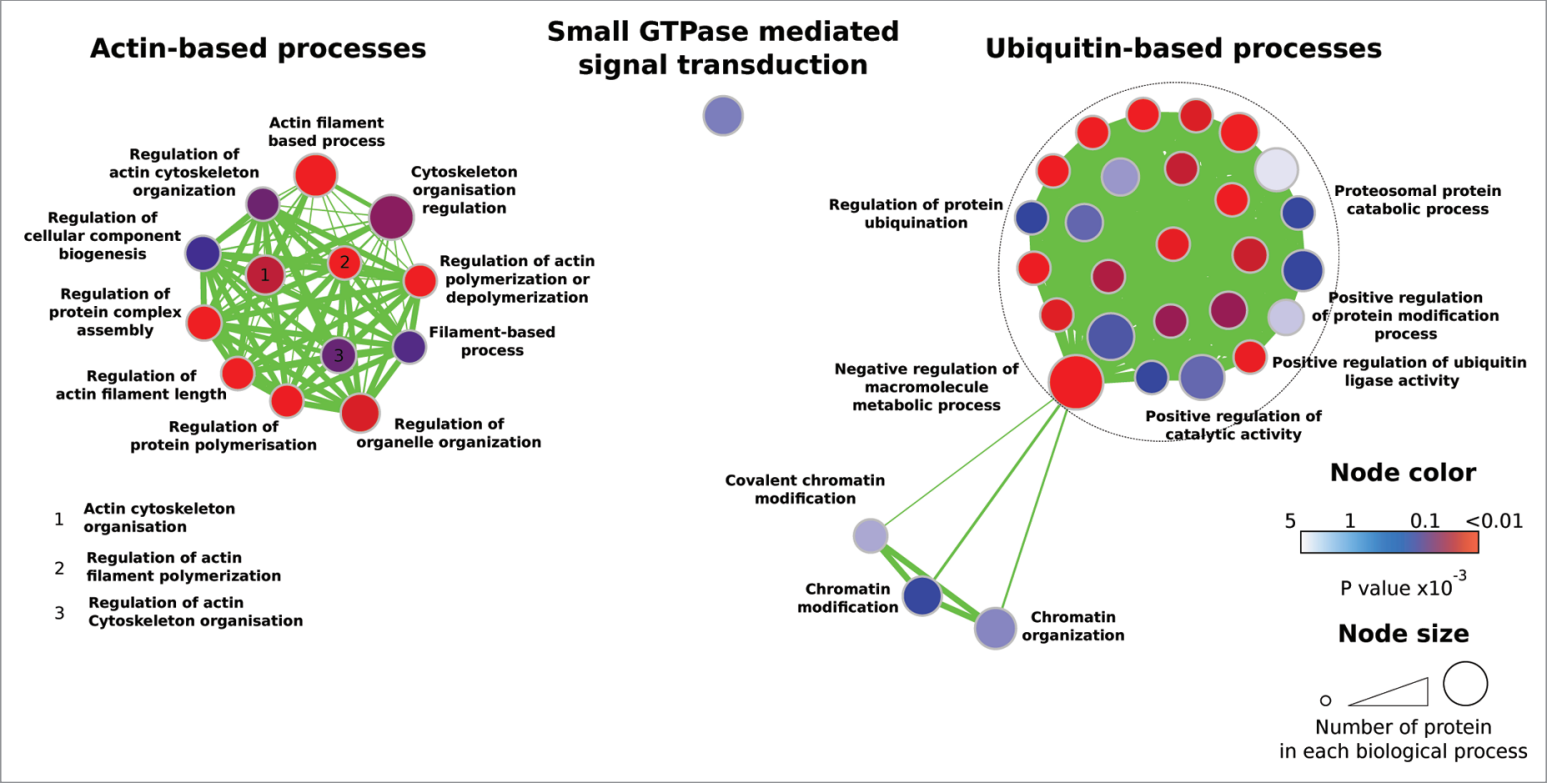
**Figure 1.** Gene Ontology analysis of IQGAP1-GFP pull-downs. Proteins enriched in the IQGAP1 pull-downs^23^ were mapped onto the GO category Biological Process (GOTERM_BP_FAT). Over-represented GO term (*P* < 0.05) were displayed as network-based enrichment maps (see Methods for details). Each node represents a GO term and each edge connects GO terms that contain at least one common protein. Node area is proportional to the number of proteins that belong to a particular GO term, and node color indicates the p value attributed to the enrichment of a particular GO term. Edge width is proportional to the similarity that exists between two GO terms. Nodes of this network were automatically organized using an algorithm that clusters nodes as a function of their connectivity and were then manually annotated.

IQGAP1 has been reported to bind directly to Rac1 and Cdc42, two small Rho family GTPases involved in the regulation of actin cytoskeleton dynamics and cell migration.^2^ IQGAP1 has been reported to prolong Cdc42 activation, by inhibiting Cdc42 intrinsic GTPase activity.^30^^,^^31^ The evidence describing IQGAP1-mediated regulation of Rac1 activity appears to be more complex suggesting a role for IQGAP1 in both positive and negative regulation of Rac1 function. Studies have reported inhibition of Rac1 activity following overexpression of an IQGAP1 construct lacking a Rac1 binding site in HEK 293T cells and following RNAi-mediated silencing of IQGAP1 in U87MG glioma cells upon serum stimulation.^18^^,^^32^ Thus, it has been proposed that, similar to Cdc42, IQGAP1-mediated activation of Rac1 may occur through inhibition of Rac1 intrinsic GTPase activity.^25^ As an alternative mechanism, the Rac1 GEF Tiam1 was shown to be recruited to IQGAP1.^33^ In contrast, we have reported a role for IQGAP1 in the negative regulation of Rac1 activity downstream of integrin α5β1 activation and/or recycling.^15^^,^^23^ Using network analyses of three published proteomic databases of integrin-associated complexes,^17^^,^^34^^,^^35^ we identified a putative link between IQGAP1, β1 integrin and Rac1 regulation (Fig. 2A) and demonstrated that, in fibroblast and osteosarcoma cells, suppression of IQGAP1 gene expression induces high, dysregulated Rac1 activity during cell spreading on fibronectin (FN) (Fig. 2B).^23^ In addition, we demonstrated that IQGAP1 silencing triggers high Rac1 activity in ovarian carcinoma cells during invasive cell migration on cell-derived matrices (CDMs).^23^ Consistent with high Rac1 activity, silencing of IQGAP1 expression in fibroblasts, osteosarcoma cells and ovarian carcinoma cells lead to unconstrained membrane protrusion and disrupted directional cell migration on fibrillar extracellular matrices.^15^^,^^23^ Mass spectrometric analysis of IQGAP1 protein complexes revealed RacGAP1 as a novel IQGAP1-binding partner, supporting a role for IQGAP1 in Rac1 deactivation.^23^ RacGAP1 was shown to be recruited by IQGAP1 to integrin activation sites in order to constrain Rac1 activity.^23^ Subsequently, RacGAP1 phosphorylation on threonine 249 by PKB/AKT, downstream of Rab-coupling protein (RCP)–dependent recycling of α5β1 integrin, was identified as a key signaling event that promoted RacGAP1 recruitment to IQGAP1 and Rac1 inactivation.^15^ Our findings clearly demonstrate that the IQGAP1-RacGAP1 interaction plays an essential role in IQGAP1-mediated inhibition of Rac1 activity. RacGAP1 is a Cdc42 GAP,^36^ and it is therefore possible that IQGAP1-mediated recruitment of RacGAP1 may also modulate Cdc42 activity. The contrasting roles reported for IQGAP1 in promoting both Rac1 activation and deactivation may be highly context-dependent and dictated by the initial cue and/or by the specificity of the IQGAP1-binding partner interaction. Nevertheless, these data suggest that IQGAP1 is an important nexus and control point for the integration of multiple signaling events that determine GTPase signaling and thus the appropriate cellular response. Interestingly, srGAP2, another Rac1-specific GAP,^37^^,^^38^ was also found to co-purify ^23^ and co-localize with IQGAP1 (data not shown) suggesting that srGAP2 could also participate in IQGAP1-mediated Rac1 regulation.

**Figure:**
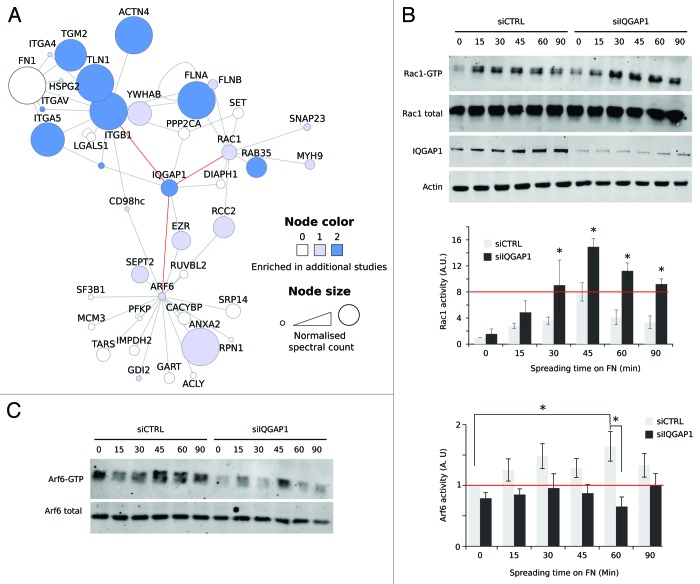
**Figure 2.** IQGAP1 is a dual regulator of Arf6 and Rac1 downstream of integrin engagement. (**A**) The network of FN-induced adhesion complexes that connects β1 integrin to Rac1 and Arf6. Proteins identified in FN-induced adhesion complexes^17^ were mapped onto a literature-curated PPI network. Each node (circle) represents a protein (labeled with gene name) and each edge (line) represents a reported interaction between two proteins. Node color indicates whether a particular protein was also identified in^35^ and/or in.^34^ Node area is proportional to the normalized spectral count of proteins identified in.^17^ Reported direct binders of β1 integrin, Rac1 and Arf6 are displayed, and red edges highlight IQGAP1 as a selected putative link between β1 integrin, Rac1 and Arf6. To allow a clear visualization of the connection between β1 integrin, Arf6 and Rac1, nodes of this network were manually organized. (**B** and **C**) To study the role of IQGAP1 in Rac1 and Arf6 activation, mouse embryonic fibroblasts were treated with control oligonucleotide (siCTRL) or siRNA targeting IQGAP1 (siIQGAP1). Rac1 (**B**) and Arf6 (**C**) activity were measured using an effector pull-down approach. To compare activation profiles between experiments and during cell spreading on FN, Rac1 and Arf6 activity were normalized to that of siCTRL cells kept in suspension (**B**, n = 4; **C**, n = 6).

IQGAP1 has additionally been reported to co-purify with the small GTPase Arf6,^18^^,^^39^ a key regulator of membrane receptor internalisation, endosomal trafficking and recycling, important processes involved in the regulation of the cell cycle, cell migration and cholesterol homeostasis.^40^ Despite the indication that IQGAP1 and Arf6 may exist in the same protein complex, there is currently no published evidence supporting a role for IQGAP1 in modulating Arf6 activity. As IQGAP1 has previously been demonstrated to be required for Arf6-mediated Rac1 activity,^18^ we speculated that IQGAP1 could coordinate Arf6 and Rac1 activities downstream of β1 integrin signaling. Indeed, further interrogation of proteomic networks, depicting integrin-associated complexes, highlighted that IQGAP1 may link β1 integrin to Arf6 function (Fig. 2A). The contribution of IQGAP1 to Arf6 activation downstream of integrin-FN engagement was therefore investigated (Fig. 2C). As previously reported, control cells spreading on FN, exhibited a gradual increase in Arf6 activity (peak at 60–90 min).^17^ FN-mediated induction of Arf6 activity was inhibited upon suppression of IQGAP1 expression (Fig. 2C), demonstrating that IQGAP1 plays a role in promoting Arf6 activation downstream of integrin-FN engagement. Mass spectrometric analysis of IQGAP1 binders identified the Arf6 GEF, HERC1 ^41^^–^^43^, as a putative novel IQGAP1- binding partner,^23^ suggesting that HERC1 could be part of the mechanism by which IQGAP1 promotes Arf6 activity downstream of integrin signaling.

In addition to Cdc42, Rac1 and Arf6, IQGAP1 has been reported to associate with and to regulate RhoA,^15^^,^^44^ a small GTPase regulating intracellular contractility, adhesion maturation, and actin dynamics.^2^ Indeed, IQGAP1 was shown to be required for EGF-mediated stimulation of RhoA activity in mammary adenocarcinoma cells.^44^ Consistently, we report that IQGAP1 is required to induce RhoA activation downstream of RCP-dependent recycling of α5β1 integrin.^15^ However, the precise mechanism of IQGAP1-mediated RhoA activation remains unknown. It has been suggested that IQGAP1 could stabilize the active GTP-bound RhoA.^44^ Additionally, mass spectrometric analysis of IQGAP1 pull-downs identified the putative RhoA GEF, PLEKHG3, as a potential novel IQGAP1 binding partner,^23^ suggesting that PLEKHG3 could be involved in IQGAP1-mediated activation of RhoA. Alternatively, as RacGAP1 has been shown to recruit the RhoA GEF, Ect2, during cytokinesis ^45^ and at cell-cell junctions,^46^ and as RacGAP1 recruitment to IQGAP1 is required to induce RhoA activation in ovarian carcinoma cells,^15^ it is possible that IQGAP1-mediated regulation of RhoA occurs via the indirect recruitment of Ect2.

IQGAP1 has also been shown to bind to and to negatively regulate the small GTPase Rap1,^47^ a regulator of integrin-mediated cell adhesion, cell–cell junction formation, establishment of cell polarity, exocytosis and cell proliferation. However, the mechanism whereby IQGAP1 suppresses Rap1 activity in cells remains unknown. In addition, IQGAP1 was found to co-purify with the small GTPase Rac2,^48^ M-Ras,^49^ RhoQ,^50^ RhoC,^51^ Rab10 and Rab5,^23^ suggesting that IQGAP1 could act upstream or downstream of these small GTPases.

The ability of IQGAP1 to recruit and regulate various small GTPases and their regulators makes IQGAP1 an ideal scaffold to orchestrate crosstalk between multiple small GTPases. Consistently, IQGAP1 was shown to be required for Arf6-mediated Rac1 activation^18^ and for Rac1-induced RhoA activity.^15^ However, how IQGAP1 temporally and spatially coordinates the small GTPases Cdc42, Rac1, RhoA, Arf6 and Rap1 is yet to be elucidated. Interestingly, a role for IQGAP1 has also been described in the modulation of processes downstream of multiple small GTPases following GTPase activation. For example, IQGAP1 regulates, downstream of Cdc42 and/or Rac1 activation, actin filament cross-linking,^52^ microtubule capture,^53^ and cadherin junctions.^54^ In addition, Cdc42 and/or RhoA activation was demonstrated to promote the recruitment of the exocyst complex to IQGAP1, resulting in matrix degradation, invadopodia formation and cell invasion.^55^ Furthermore, IQGAP1 was found to recruit multiple small GTPase effectors including PAK6,^56^ N-WASP,^57^^,^^58^ several sub-units of the Arp2/3 complex^23^^,^^57^^,^^58^ and the formin mDia1.^59^ Therefore, IQGAP1 is not only a modulator of GTPase activity but can also be described as a small GTPase effector.^24^^,^^25^

As IQGAP1 modulates the activity of multiple small GTPases, participates in small GTPase crosstalk and regulates processes downstream of activated small GTPases, we propose that IQGAP1 acts as a small GTPase scaffolding platform, recruiting and/or regulating small GTPases, small GTPase regulators and effectors to orchestrate cell behavior (Fig. 3). A fundamental question to address will be to determine whether these molecules are recruited to IQGAP1 simultaneously or at discrete subcellular locations and/or time, and to identify the signaling events (integrins, syndecans, growth factor receptors) and/or the post-translational modifications that dictate the differential recruitment of these GTPase regulators and effectors to IQGAP1.

**Figure:**
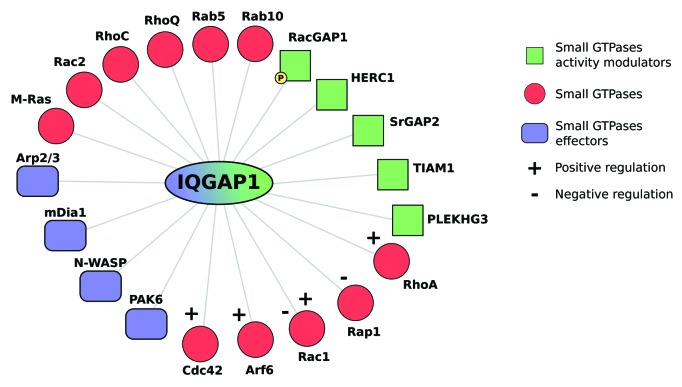
**Figure 3.** IQGAP1 recruits small GTPases, small GTPase regulators and small GTPase effectors to regulate cell behavior. Schematic representation of small GTPases, small GTPase regulators and small GTPase effectors known to co-purify with IQGAP1. Small GTPases are highlighted in red, small GTPase regulators in green and small GTPase effectors in blue.

Our understanding of small GTPase crosstalk is principally limited by the difficulty of simultaneously measuring the activation state of multiple small GTPases. However, as there is increasing evidence that small GTPases can dynamically regulate each other, we envisage that small GTPases are part of a broad and complex regulatory network, and believe that understanding this GTPase network will enable the modeling of complex cellular behavior. We predict that this network will contain several key nodes, such as IQGAP1, that will connect multiple small GTPases and will be responsible for small GTPase crosstalk. Considering the importance of IQGAP1 in physiological processes such as cell migration and in disease,^15^^,^^23^^,^^26^^,^^60^ it is likely that these other “signalling hubs” will constitute equally critical regulators of cell behavior and valuable therapeutic targets.

In order to identify other putative signaling hubs, we constructed a global small GTPase network in silico. 144 out of the 162 known small GTPases were mapped onto literature-based protein-protein interaction (PPI) databases (excluding 18 small GTPases not found in the databases) and the known direct binding partners of these GTPases were selected. Intriguingly, the amalgamation of the known small GTPases and GTPase interactors resulted in one single network containing 1310 nodes and 11110 edges (Fig. 4). This network demonstrates that even with our limited knowledge of some of the small GTPases and their binding partners, every mapped small GTPase interacts directly or indirectly via common regulators with another small GTPase. This observation further reinforces the notion that small GTPases assemble a broad regulatory network.^8^ Network stress analysis, a measure of the number of shortest paths passing through a node, (i.e., if the shortest path between node A and node B crosses through node C, then a stress value is assigned to node C) was then used to highlight putative key scaffolds within the GTPase network. The degree of network stress applied to a particular node, i.e., the number of shortest paths passing through that node, highlights the importance of that node to integrate and transmit information within a network and therefore to act as a signaling hub. Using this network stress analysis IQGAP1 was identified as one major node within the small GTPase network as well as many other proteins including GRB2, FLNA, PIK3R1, MDM2, EP300, CTNNB1 (Fig. 4; Table S1). The identification of IQGAP1 as a regulatory hub strengthens the hypothesis that global analysis of the GTPase network can be used to short-list proteins involved in small GTPase crosstalk. However, this network remains non-functional as it does not take into account the activation states of the various small GTPases. Indeed, small GTPases act as molecular switches that recruit different effectors in function of their activation state. Therefore, mapping and understanding small GTPase crosstalk will require the assembly of activation-state specific networks. Ultimately, it is likely that mathematical modeling and systems-based approaches will be required to unravel the dynamic structure of the GTPase network and to delineate positive and negative crosstalk pathways. These activation state-specific small GTPase networks will be central to an understanding of how small GTPase activities are orchestrated to transduce the signaling flux responsible for the regulation of complex physiological processes such as cell migration.

**Figure:**
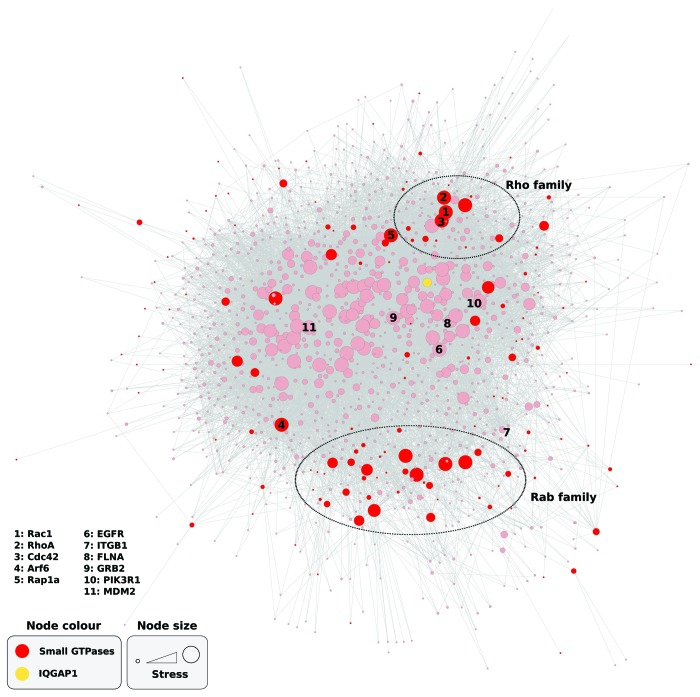
**Figure 4.** IQGAP1 is a key node within the small GTPase network. To construct a small GTPase network, 144 out of the 162 known small GTPases were mapped onto literature-based protein-protein interaction (PPI) databases (18 small GTPases not found in the databases) and their known direct binding partners were selected. Each node represents a protein and each edge represents a reported interaction between 2 proteins. Nodes of this network were automatically organized using an algorithm that clusters nodes as a function of their connectivity. Small GTPases are highlighted in Red and IQGAP1 in yellow. Node size is proportional to the network topological parameter Stress. The stress of a node n is the number of shortest paths passing through n. A node has a high stress if it is traversed by a high number of shortest paths. Some nodes of interest were manually labeled.

## Material and Methods

### Cell culture

Wild-type mouse embryonic fibroblasts (MEFs) were maintained in Dulbecco's modified Eagle’s medium (DMEM; Sigma-Aldrich) supplemented with 10% (vol/vol) fetal calf serum (FCS; Biosera) and 20 U/ml mouse interferon γ (Sigma-Aldrich) at 33 °C, 5% (vol/vol) CO_2_.

### Reagents

Primary antibodies used in this study were rabbit anti-human IQGAP1 (H-109; Santa Cruz Biotechnology), mouse anti-human Rac1 (BD Biosciences), mouse anti- human actin (AC-40; Sigma-Aldrich) and mouse anti-human Arf6 (ARFAG, Sigma-Aldrich). Secondary antibodies Alexa- Fluor-680-conjugated or Alexa-Fluor-800-conjugated, used for western blotting, were provided by Invitrogen.

### siRNA knockdown

The expression of proteins of interest was reduced using 100 nM ON- TARGETplus modified siRNA (Dharmacon, Thermo Fisher) and Lipofectamine 2000 (Invitrogen) according to the manufacturer’s instructions. Two rounds of transfection were necessary to achieve efficient knock-down. The ON- TARGETplus non-targeting siRNA (Dharmacon) was used as a negative control. The siRNA oligo used to knock-down mouse IQGAP1 was GGACAAUGCCGUUAGCGAA (Dharmacon, J-040589–12) Levels of knockdown were verified by western blotting for every experiment.

### Rac1 and Arf6 activity assay

Active Rac1 and active Arf6 were affinity purified from cell lysates using an effector pull-down approach with GST-PAK beads and GST-GGA3 beads, respectively. Cells were serum starved and maintained in suspension for 1 h before plating on 10 μg/ml FN-coated dishes. Cells were lysed in ice-cold GTPase-specific lysis buffer [Rac1: 20 mM Hepes, pH 7.5, 1% (vol/vol) Igepal, 0.5% (wt/vol) sodium deoxycholate, 140 mM NaCl, 4 mM EDTA, 4 mM EGTA, 10% (vol/vol) glycerol); Arf6: 50 mM TRIS-HCl, pH 7.5, 1% (vol/vol) Triton X-100, 150 mM NaCl, 10% (vol/vol) glycerol, 10 mM MgCl 2, 0.5% (wt/vol) sodium deoxycholate, 0.1% (wt/vol) SDS, 1 mM Na3VO4, and protease inhibitors] at different time points, and lysates were clarified by centrifugation at 12,000 g, 4 °C, for 1 min. Lysates were then incubated with GST-PAK beads(Rac1 assay) beads or GST-GGA3 beads (Arf6 assay) for 1 h at 4 °C. Beads were washed three times with ice-cold lysis buffer (Rac1 assay) or with ice-cold Arf6 washing buffer (50 mM TRIS-HCl, pH 7.5, 1% (vol/vol) Igepal, 100 mM NaCl, 10% (vol/vol) glycerol, 2 mM MgCl2) and active GTPase was eluted by addition of Laemmli reducing sample buffer. Samples were then resolved by SDS-PAGE and analyzed by western blotting.

### SDS-PAGE and quantitative western blotting

Protein extracts were separated under denaturing conditions by SDS-PAGE (4– 12% Bis-Tris gels; Invitrogen) and transferred to nitrocellulose membrane. Membranes were blocked overnight at 4 ˚C with blocking buffer (Sigma-Aldrich) and then incubated with the appropriate primary antibody diluted in blocking buffer (Sigma-Aldrich) for 2 h. Membranes were washed with PBS and then incubated with the appropriate fluorophore-conjugated secondary antibody diluted 1:5000 in blocking buffer for 30 min. Membranes were washed in the dark and then scanned using an Odyssey infrared imaging system (LI-COR Biosciences). Band intensity was determined by digital densitometric analysis using Odyssey software (version 2.1; LI-COR Biosciences).

### Mass spectrometric data analysis

Details of the IQGAP1 data set mentioned in this study are available in reference 23 Data were also deposited in the PRIDE database (http://www.ebi.ac.uk/pride)^61^ under accession numbers 26836–26850. Gene Ontology analysis was performed using DAVID (version 6.7)^62^ using the Gene Ontology category “Biological Process” (GOTERM_BP_FAT). Gene Ontology terms over-represented in the IQGAP1 data set (*P* < 0.05) were clustered and represented as network-based enrichment maps using the Cytoscape^63^ plug-in Enrichment Map (v1.2) (Threshold of, p value, 0.005; FDR. 0.1; overlap coefficient, 0.5).^64^ PPI network analysis was performed using Cytoscape (version 2.8.1).^63^ Proteins belonging to the Ras superfamily were mapped onto a merged human interactome consisting of PPIs reported in the Protein Interaction Network Analysis platform Homo sapiens network (release date 28th June 2011)^65^ and literature-curated databases of integrin adhesion-associated proteins.^66^^,^^67^ Network Stress analysis or stress centrality (stress centrality of a node n is the number of shortest paths passing through n) was performed within Cytoscape using the NetworkAnalyzer plugin.

### Statistical analysis

Statistical analyses were performed using the Student’s *t* test (unpaired, two-tailed, unequal variance). P values are indicated by an asterisk in the figure legends.

## Supplementary Material

Additional material

## Abbreviations:

GAPs: GTPase-activating proteins
GEFs: guanine nucleotide exchange factors
PPI: protein-protein interaction
FN: fibronectin
MEFs: mouse embryonic fibroblasts
CDMs: cell-derived matrices
