# Supplementary material for: IQGAP1 is a key node within the small GTPase network
Source: Small GTPases. 2013 Dec 19;4(4):199–207. doi: 10.4161/sgtp.27451 (PMC4011815; doi:10.4161/sgtp.27451)
Supplement: Additional material [file sgtp-4-199-s01.pdf]

Landes Bioscience www.landesbioscience.com

Supplemental Material to:

Guillaume Jacquemet and Martin J Humphries

IQGAP1 is a key node within the small GTPase network

Small GTPases 2013; 4(4)
http://dx.doi.org/10.4161/sgtp.27451

http://www.landesbioscience.com/journals/smallgtpases/article/27451

Uniprot ID	Gene Name	Stress	small GTPase
P0CG48	UBC	5278432	
P63000	RAC1	460586	yes
P60953	CDC42	413700	yes
P62993	GRB2	384118	
Q15717	ELAVL1	380650	
P63104	YWHAZ	334616	
P61586	RHOA	294128	yes
P01112	HRAS	285024	yes
P12931	SRC	248074	
P62330	ARF6	221198	yes
Q13618	CUL3	181456	
P04637	p53	174526	
P00533	EGFR	171832	
Q9Y4K3	TRAF6	169270	
P40337	VHL	146196	
P61956	SUMO2	145712	
P01106	MYC	141474	
P20338	RAB4A	134056	yes
P27986	PIK3R1	133558	
P61981	YWHAG	125702	
Q9Y6K9	IKBKG	117600	
P62158	CALM1	115724	
P04049	RAF1	104594	
P62834	RAP1A	99222	yes
Q15796	SMAD2	98832	
P17252	PRKCA	96880	
P68400	CSNK2A1	96310	
Q00987	MDM2	95036	
P20339	RAB5A	93092	yes
Q13485	SMAD4	92748	
P84077	ARF1	91868	yes
P51159	RAB27A	91638	yes
Q15004	KIAA0101	89976	
Q14164	IKBKE	89544	
P62826	RAN	87802	yes
Q09472	EP300	85588	
Q04917	YWHAH	85234	
P32121	ARRB2	84094	
P63165	SUMO1	83626	
P36897	TGFBR1	82994	
Q12933	TRAF2	80114	
P08670	VIM	79226	
P27348	YWHAQ	77138	
P60709	ACTB	76862	
P84022	SMAD3	76682	
P21333	FLNA	73850	
P62820	RAB1A	72580	yes
P04183	TK1	71488	
Q92905	COPS5	71432	
P10114	RAP2A	70506	yes

Q13153	PAK1	70142	
P35222	CTNNB1	70142	
Q13526	PIN1	69398	
P17612	PRKACA	67494	
Q9UI14	RABAC1	67068	
Q93034	CUL5	66354	
P19174	PLCG1	66118	
Q13547	HDAC1	65962	
P31946	YWHAB	65754	
P62491	RAB11A	64856	yes
P31749	AKT1	63934	
P07910	HNRNPC	63534	
P06241	FYN	62566	
P61224	RAP1B	61356	yes
P55040	GEM	61148	yes
Q15858	SCN9A	60356	
Q00610	CLTC	59152	
P00519	ABL1	58702	
P63279	UBE2I	58444	
P51149	RAB7A	57482	yes
Q16637	SMN1	56958	
P20340	RAB6A	56628	yes
Q05397	PTK2	56400	
P42574	CASP3	56390	
P11233	RALA	55688	yes
P49407	ARRB1	55332	
P40763	STAT3	55328	
Q15811	ITSN1	54680	
P15311	VIL2	54112	
Q13616	CUL1	52602	
P10301	RRAS	52298	yes
Q9Y265	RUVBL1	51968	
Q05513	PRKCZ	51292	
P46108	CRK	50272	
P26641	EEF1G	50156	
P63167	DYNLL1	49870	
Q9NRC8	SIRT7	49452	
P41743	PRKCI	49396	
P11274	BCR	49008	
P62258	YWHAE	48770	
P61026	RAB10	48138	yes
Q9HCE7	SMURF1	47900	
P22681	CBL	47094	
O15162	PLSCR1	46780	
P46940	IQGAP1	46570	
P03372	ESR1	46304	
P30480	HLA-B	46054	
P27361	MAPK3	46020	
P49703	ARL4D	45772	yes
P06493	CDK1	44666	
Q01105	SET	44628	
P08107	HSPA1A	43944	
P16333	NCK1	43500	

P48023	FASLG	43452	
P07355	ANXA2	43322	
Q15286	RAB35	43064	yes
Q96PM5	RCHY1	42680	
P06702	S100A9	41996	
P62070	RRAS2	40924	yes
P63244	GNB2L1	40390	
P60763	RAC3	40258	yes
Q86UR5	RIMS1	39818	
P31947	SFN	39490	
Q96B97	SH3KBP1	39368	
P60520	GABARAPL2	39138	
Q92769	HDAC2	38900	
Q9HAU4	SMURF2	38814	
Q9Y230	RUVBL2	38530	
Q8TAI7	RHEBL1	38178	yes
P23508	MCC	37908	
P52292	KPNA2	37590	
P36406	TRIM23	37588	yes
P11171	EPB41	37246	
Q15843	NEDD8	37160	
Q13177	PAK2	36838	
P61019	RAB2A	36266	yes
Q9P0S9	TMEM14C	36032	
P30153	PPP2R1A	35990	
P20337	RAB3B	35906	yes
P54253	ATXN1	35644	
P55042	RRAD	35564	yes
Q03135	CAV1	35340	
P45983	MAPK8	35274	
Q01844	EWSR1	35252	
Q96PU5	NEDD4L	35226	
Q92993	KAT5	35168	
P49023	PXN	34486	
P61006	RAB8A	34360	yes
P04150	NR3C1	34306	
O00212	RHOD	34178	yes
P05556	ITGB1	33450	
O14641	DVL2	33348	
P25445	FAS	33342	
Q8TBB1	LNX1	32172	
Q9Y478	PRKAB1	32006	
Q9UQL6	HDAC5	31932	
Q05655	PRKCD	31884	
P20936	RASA1	31654	
Q9UQ26	RIMS2	31594	
Q99558	MAP3K14	31480	
Q04695	KRT17	31264	
P46934	NEDD4	31156	
Q9H0U4	RAB1B	30932	yes
Q96GG9	DCUN1D1	30170	
O14818	PSMA7	30104	
Q5T3J3	LRIF1	30042	

P01111	NRAS	29436	yes
P61077	UBE2D3	29144	
Q7L5N1	COPS6	29104	
P05109	S100A8	28846	
O15085	ARHGEF11	28718	
Q9NQC3	RTN4	28690	
Q9H0T7	RAB17	28496	yes
Q92947	GCDH	28452	
P12956	XRCC6	28434	
P68366	TUBA4A	28320	
Q07889	SOS1	28122	
P15498	VAV1	28074	
P25963	NFKBIA	27992	
P57735	RAB25	27162	yes
P42336	PIK3CA	26998	
O00238	BMPR1B	26628	
O43924	PDE6D	26248	
P68133	ACTA1	26054	
Q96S21	RAB40C	26028	yes
O95376	ARIH2	25928	
Q6IPS9	EEF1A1	25730	
Q99816	TSG101	25718	
P62873	GNB1	25624	
P06748	NPM1	25356	
Q13393	PLD1	25316	
Q14203	DCTN1	25280	
Q15382	RHEB	24688	yes
Q9NRR5	UBQLN4	24482	
P06239	LCK	24458	
Q92963	RIT1	24310	yes
Q9UGL1	KDM5B	24060	
Q9Y3P9	RABGAP1	24024	
Q9BU20	RSG1	23994	yes
P40692	MLH1	23994	
P43487	RANBP1	23650	
Q9BVJ6	UTP14A	23236	
P02792	FTL	23128	
P67775	PPP2CA	23058	
P00491	PNP	22846	
Q5R372	RABGAP1L	22580	
Q99750	MDFI	22580	
Q13310	PABPC4	22484	
Q13263	TRIM28	22444	
P10275	AR	22440	
P62745	RHOB	22434	yes
O15151	MDM4	22354	
P49815	TSC2	22344	
P56945	BCAR1	22060	
P51116	FXR2	21976	
P31150	GDI1	21072	
O43150	DDEF2	21036	
O14939	PLD2	20880	
P25786	PSMA1	20768	

Q14974	KPNB1	20668	
Q16891	IMMT	20540	
P51617	IRAK1	20526	
O60739	EIF1B	20500	
Q9H0F7	ARL6	20318	yes
O14964	HGS	20276	
Q9H8T0	AKTIP	20262	
P52565	ARHGDIA	20174	
P14373	TRIM27	20060	
Q02750	MAP2K1	20020	
P15153	RAC2	19962	yes
P18754	RCC1	19908	
P42768	WAS	19836	
Q70SY1	CREB3L2	19834	
Q13233	MAP3K1	19830	
Q08379	GOLGA2	19786	
Q9BZG1	RAB34	19754	yes
Q15797	SMAD1	19716	
P61106	RAB14	19418	yes
P14618	PKM	19342	
P50395	GDI2	19320	
Q9H0H5	RACGAP1	19282	
Q9BSI4	TINF2	19248	
Q71U36	TUBA1A	19242	
P49768	PSEN1	19094	
Q9NXU5	ARL15	19038	yes
O15350	TP73	19026	
P42224	STAT1	18950	
P51532	SMARCA4	18802	
Q9Y2H9	MAST1	18766	
P19438	TNFRSF1A	18748	
P25205	MCM3	18666	
Q9UKE5	TNIK	18648	
P35240	NF2	18578	
P20336	RAB3A	18392	yes
P51148	RAB5C	18354	yes
P54259	ATN1	18326	
P11234	RALB	18300	yes
Q13114	TRAF3	18268	
O43639	NCK2	18234	
P23246	SFPQ	18186	
P05771	PRKCB	18134	
P78559	MAP1A	17892	
P68104	EEF1A1	17818	
P36405	ARL3	17754	yes
Q9Y2J0	RPH3A	17532	
Q14204	DYNC1H1	17526	
O14980	XPO1	17470	
Q9ULH1	DDEF1	17240	
Q9HB71	CACYBP	17190	
P18206	VCL	17160	
P10415	BCL2	17150	
Q15907	RAB11B	17046	yes

P18031	PTPN1	16840	
O00459	PIK3R2	16812	
P16104	H2AFX	16730	
Q5UIP0	RIF1	16684	
P07737	PFN1	16640	
P04406	GAPDH	16542	
Q969H4	CNKSR1	16498	
P61587	RND3	16442	yes
Q13432	UNC119	16392	
O15211	RGL2	16328	
Q13347	EIF3I	16136	
P40616	ARL1	16030	yes
Q13137	CALCOCO2	15990	
P17081	RHOQ	15860	yes
Q08211	DHX9	15810	
P04629	NTRK1	15790	
P21980	TGM2	15742	
P52272	HNRNPM	15728	
P35579	MYH9	15622	
Q05516	ZBTB16	15574	
Q9H0E2	TOLLIP	15414	
Q15276	RABEP1	15364	
P29475	NOS1	15230	
P06213	INSR	14912	
Q9H2M9	RAB3GAP2	14692	
P46379	BAG6	14684	
P28340	POLD1	14646	
Q14289	PTK2B	14632	
P61020	RAB5B	14438	yes
Q9ULW5	RAB26	14406	yes
P12268	IMPDH2	14252	
Q9UKG1	APPL1	14166	
P50502	ST13	14048	
P30304	CDC25A	14036	
P42345	MTOR	14026	
P10276	RARA	13896	
O60216	RAD21	13890	
P04179	SOD2	13746	
Q92831	KAT2B	13682	
P51153	RAB13	13640	yes
Q07157	TJP1	13610	
P55210	CASP7	13400	
Q16658	FSCN1	13344	
Q16659	MAPK6	13330	
Q9NS23	RASSF1	13288	
Q14697	GANAB	13288	
P27695	APEX1	13278	
O95477	ABCA1	13266	
O14807	MRAS	13160	yes
P05129	PRKCG	13134	
P55786	NPEPPS	13114	
Q15052	ARHGEF6	13110	
Q9NR12	PDLIM7	13050	

P55209	NAP1L1	13000	
P98170	XIAP	12896	
P49792	RANBP2	12876	
P05161	ISG15	12818	
P46527	CDKN1B	12770	
P19338	NCL	12750	
P05783	KRT18	12726	
P52306	RAP1GDS1	12666	
O00329	PIK3CD	12628	
P15056	BRAF	12524	
P19793	RXRA	12370	
P04844	RPN2	12362	
O94955	RHOBTB3	12312	
P60228	EIF3E	12284	
Q9UBF6	RNF7	12256	
Q9BWF2	TRAIP	12228	
Q9Y678	COPG1	12014	
Q13671	RIN1	11938	
P08134	RHOC	11906	yes
P01023	A2M	11870	
Q15370	TCEB2	11786	
P28074	PSMB5	11778	
O15399	GRIN2D	11754	
P00505	GOT2	11736	
Q13885	TUBB2A	11730	
O95257	GADD45G	11728	
Q99578	RIT2	11680	yes
O14936	CASK	11332	
P61225	RAP2B	11186	yes
Q9UJ41	RABGEF1	11040	
P21912	SDHB	10984	
P55196	MLLT4	10976	
Q07912	TNK2	10894	
O60828	PQBP1	10870	
P07437	TUBB	10750	
Q8IUC4	RHPN2	10722	
P41220	RGS2	10692	
Q9Y5M8	SRPRB	10672	
P11586	MTHFD1	10656	
Q9NSC2	SALL1	10650	
Q99418	CYTH2	10622	
Q15438	CYTH1	10498	
Q9P258	RCC2	10438	
Q9Y5K6	CD2AP	10376	
Q16513	PKN2	10360	
Q13129	RLF	10344	
Q9NRY4	ARHGAP35	10284	
P49810	PSEN2	10176	
Q9Y2Q3	GSTK1	10154	
P12830	CDH1	10140	
Q9BV36	MLPH	10130	
P62256	UBE2H	10094	
P53611	RABGGTB	10078	

P63096	GNAI1	9952	
Q9UNE2	RPH3AL	9946	
Q9P2H0	KIAA1377	9886	
Q93062	RBPMS	9884	
Q92930	RAB8B	9844	yes
Q9NRD5	PICK1	9784	
Q9Y2Y0	ARL2BP	9704	
Q15645	TRIP13	9690	
O00471	EXOC5	9668	
Q96L34	MARK4	9580	
P47224	RABIF	9574	
Q96EV8	DTNBP1	9566	
P24385	CCND1	9546	
Q13315	ATM	9498	
Q9Y547	HSPB11	9466	
Q96EY1	DNAJA3	9456	
Q8IYI6	EXOC8	9424	
Q96RU3	FNBP1	9356	
P26038	MSN	9284	
P49790	NUP153	9272	
P47736	RAP1GAP	9116	
P51151	RAB9A	9108	yes
O75533	SF3B1	9042	
B0QYK1	EWSR1	9034	
P49354	FNTA	9030	
Q15369	TCEB1	9020	
Q02641	CACNB1	9020	
Q13490	BIRC2	9008	
Q9Y2X7	GIT1	8886	
Q96LR5	UBE2E2	8804	
Q9HAV4	XPO5	8780	
O00299	CLIC1	8776	
P01116	KRAS	8740	yes
O95989	NUDT3	8690	
P10398	ARAF	8684	
P33176	KIF5B	8650	
Q96GM8	TOE1	8538	
P53618	COPB1	8532	
Q9NZN5	ARHGEF12	8512	
P14598	NCF1	8512	
Q92922	SMARCC1	8506	
P56537	EIF6	8490	
Q12967	RALGDS	8322	
Q07960	ARHGAP1	8304	
Q92558	WASF1	8272	
P52735	VAV2	8272	
P58107	EPPK1	8260	
O43187	IRAK2	8256	
Q01968	OCRL	8192	
Q9NZ52	GGA3	8174	
Q8NFJ9	BBS1	8166	
Q00005	PPP2R2B	8124	
Q9UKW4	VAV3	8080	

P23443	RPS6KB1	8068	
Q8TAQ2	SMARCC2	8060	
O14924	RGS12	8044	
O95782	AP2A1	8044	
P31689	DNAJA1	8004	
O43157	PLXNB1	7866	
Q9Y266	NUDC	7846	
Q9GZV4	EIF5A2	7826	
Q04771	ACVR1	7818	
P38936	CDKN1A	7720	
Q7Z6J0	SH3RF1	7718	
P35520	CBS	7706	
Q9Y6R4	MAP3K4	7698	
Q13332	PTPRS	7686	
P22392	NME2	7672	
P19525	EIF2AK2	7634	
P07711	CTSL1	7614	
Q13009	TIAM1	7594	
P30307	CDC25C	7582	
Q13555	CAMK2G	7560	
Q14155	ARHGEF7	7522	
O60664	PLIN3	7478	
P84085	ARF5	7452	yes
P50749	RASSF2	7286	
Q92574	TSC1	7216	
P10588	NR2F6	7208	
Q9UMN6	KMT2B	7198	
Q5SU16	TUBB	7178	
Q8N1F7	NUP93	7162	
Q9Y2W2	WBP11	7156	
Q9UJY4	GGA2	7140	
Q9UQB8	BAIAP2	7114	
P42680	TEC	7096	
Q92597	NDRG1	7090	
P55735	SEC13	7068	
P09382	LGALS1	7060	
O15066	KIF3B	7050	
P07951	TPM2	7030	
O60725	ICMT	7030	
O95757	HSPA4L	6974	
Q99836	MYD88	6936	
Q05586	GRIN1	6910	
E5KNY5	LRPPRC	6886	
Q99832	CCT7	6836	
P51571	SSR4	6834	
Q7Z569	BRAP	6742	
P50281	MMP14	6728	
Q16584	MAP3K11	6726	
Q9NPB6	PARD6A	6694	
O00560	SDCBP	6666	
Q9P107	GMIP	6666	
Q9NVJ2	ARL8B	6654	yes
Q8IUD2	ERC1	6640	

Q9BYG5	PARD6B	6588	
Q15311	RALBP1	6582	
Q96FW1	OTUB1	6566	
Q6WKZ4	RAB11FIP1	6520	
Q99933	BAG1	6504	
Q01813	PFKP	6480	
Q9UQ13	SHOC2	6480	
Q8IWV7	UBR1	6478	
O95793	STAU1	6476	
Q9H8Y8	GORASP2	6466	
Q6WCQ1	MPRIP	6464	
Q99614	TTC1	6426	
P18124	RPL7	6426	
Q9UET6	FTSJ1	6380	
Q12824	SMARCB1	6378	
A7KAX9	ARHGAP32	6376	
P45973	CBX5	6372	
Q8WWW0	RASSF5	6366	
O14965	AURKA	6336	
P53365	ARFIP2	6320	
Q13158	FADD	6300	
Q8NC60	NOA1	6274	
P81274	GPSM2	6264	
P49356	FNTB	6240	
P52566	ARHGDIB	6234	
Q9Y6X2	PIAS3	6178	
P07814	EPRS	6176	
Q9UJY5	GGA1	6150	
Q8TEW0	PARD3	6114	
Q9NR09	BIRC6	6110	
P49715	CEBPA	6036	
P48507	GCLM	6032	
Q15363	TMED2	6016	
O00217	NDUFS8	6016	
A5PKW4	PSD	5956	
O43261	DLEU1	5954	
P62273	RPS29	5876	
Q9BU61	NDUFAF3	5866	
P49755	TMED10	5860	
P51531	SMARCA2	5852	
P07196	NEFL	5802	
Q8TDJ6	DMXL2	5744	
P46459	NSF	5720	
P16220	CREB1	5714	
Q9BTW9	TBCD	5704	
Q15642	TRIP10	5688	
Q15653	NFKBIB	5628	
P13569	CFTR	5552	
Q9UKT9	IKZF3	5522	
P84095	RHOG	5520	yes
Q15042	RAB3GAP1	5520	
Q92734	TFG	5504	
P21246	PTN	5470	

Q9UIW2	PLXNA1	5368	
Q8NC51	SERBP1	5364	
Q12904	AIMP1	5364	
Q9BT78	COPS4	5344	
Q13541	EIF4EBP1	5328	
P83916	CBX1	5324	
P26639	TARS	5300	
O75390	CS	5296	
P22695	UQCRC2	5252	
Q14185	DOCK1	5234	
P18085	ARF4	5226	yes
P49321	NASP	5202	
Q13464	ROCK1	5194	
O95716	RAB3D	5194	yes
Q5U651	RASIP1	5188	
P55060	CSE1L	5174	
Q9UPA5	BSN	5160	
Q92888	ARHGEF1	5150	
P02786	TFRC	5128	
P39656	DDOST	5108	
P08195	SLC3A2	5098	
P43246	MSH2	5090	
Q01201	RELB	5066	
P50213	IDH3A	5052	
Q96ST3	SIN3A	5034	
Q9Y6G9	DYNC1LI1	5026	
P42704	LRPPRC	5000	
Q92974	ARHGEF2	4984	
P13498	CYBA	4936	
Q92945	KHSRP	4900	
Q96AX2	RAB37	4870	yes
P60604	UBE2G2	4864	
Q53EZ4	CEP55	4796	
Q8N4C8	MINK1	4794	
Q96N67	DOCK7	4770	
P04899	GNAI2	4752	
Q53G59	KLHL12	4746	
Q92730	RND1	4684	yes
Q9NVI1	FANCI	4672	
Q9UBU9	NXF1	4640	
O00189	AP4M1	4620	
Q15075	EEA1	4604	
Q9NRW1	RAB6B	4602	yes
Q9UL26	RAB22A	4580	yes
O60271	SPAG9	4566	
O43592	XPOT	4562	
O75821	EIF3G	4532	
P07384	CAPN1	4532	
O15031	PLXNB2	4514	
Q9UPT5	EXOC7	4510	
P09661	SNRPA1	4492	
Q14194	CRMP1	4488	
Q9Y5K5	UCHL5	4478	

Q12846	STX4	4460	
Q09013	DMPK	4454	
O95197	RTN3	4426	
Q9UL19	RARRES3	4422	
O43566	RGS14	4412	
Q7L2J0	MEPCE	4372	
Q8NDX1	PSD4	4354	
P02749	APOH	4350	
P56134	ATP5J2	4342	
P04843	RPN1	4340	
Q96HA1	POM121	4330	
P42684	ABL2	4314	
Q03113	GNA12	4298	
Q96A65	EXOC4	4296	
Q8N6T3	ARFGAP1	4274	
Q86Y07	VRK2	4268	
O14578	CIT	4258	
Q13636	RAB31	4252	yes
Q14457	BECN1	4228	
O60496	DOK2	4196	
Q01664	TFAP4	4186	
P61204	ARF3	4182	yes
P24386	CHM	4160	
Q9UKR5	C14orf1	4148	
Q86YS3	RAB11FIP4	4110	
P30305	CDC25B	4102	
Q9HD15	SRA1	4100	
P46060	RANGAP1	4084	
Q96CV9	OPTN	4076	
Q04724	TLE1	4068	
Q13185	CBX3	4062	
P19878	NCF2	4042	
Q9NYB9	ABI2	4042	
Q9UPN3	MACF1	4030	
P51665	PSMD7	4028	
Q96T51	RUFY1	4006	
P25685	DNAJB1	4002	
Q8NEB9	PIK3C3	3988	
P53801	PTTG1IP	3982	
Q9H5N1	RABEP2	3982	
Q9P212	PLCE1	3964	
Q13574	DGKZ	3926	
O75674	TOM1L1	3880	
P32969	RPL9	3852	
Q15459	SF3A1	3844	
O00401	WASL	3838	
Q9NYS0	NKIRAS1	3828	yes
Q12982	BNIP2	3820	
P20749	BCL3	3820	
O14617	AP3D1	3800	
O00203	AP3B1	3762	
Q12809	KCNH2	3758	
Q8N8R7	ARL14EP	3746	

P29323	EPHB2	3726	
Q8TB24	RIN3	3712	
Q9UBF2	COPG2	3712	
O43543	XRCC2	3700	
O60610	DIAPH1	3690	
Q9UNA1	ARHGAP26	3686	
P51813	BMX	3680	
P11177	PDHB	3676	
Q15019	SEPT2	3670	
Q9Y2A7	NCKAP1	3664	
Q9NP72	RAB18	3662	yes
Q86UP2	KTN1	3662	
P30419	NMT1	3642	
P78357	CNTNAP1	3628	
Q16623	STX1A	3622	
Q9H8V3	ECT2	3598	
Q02779	MAP3K10	3590	
P04839	CYBB	3590	
O15056	SYNJ2	3568	
Q92973	TNPO1	3556	
Q96BK5	PINX1	3544	
O75369	FLNB	3544	
P18887	XRCC1	3540	
P54577	YARS	3528	
O75914	PAK3	3528	
O00410	IPO5	3528	
P52198	RND2	3494	yes
Q9UHV9	PFDN2	3480	
P53367	ARFIP1	3476	
Q9H0S4	DDX47	3432	
P33316	DUT	3426	
Q9UI26	IPO11	3422	
P83876	TXNL4A	3420	
P28799	GRN	3412	
Q9NYR9	NKIRAS2	3404	yes
Q9UN37	VPS4A	3400	
Q9UM73	ALK	3364	
Q9Y613	FHOD1	3356	
Q8TDZ2	MICAL1	3342	
Q9BZ29	DOCK9	3322	
Q9GZM8	NDEL1	3314	
P35268	RPL22	3294	
O75962	TRIO	3292	
P37837	TALDO1	3278	
O96013	PAK4	3264	
Q8NF50	DOCK8	3224	
P10911	MCF2	3218	
O14579	COPE	3182	
P62829	RPL23	3166	
O95273	CCNDBP1	3162	
P19022	CDH2	3142	
Q70J99	UNC13D	3140	
Q00587	CDC42EP1	3092	

P61970	NUTF2	3088	
Q02535	ID3	3086	
Q16555	DPYSL2	3082	
Q9UI30	TRMT112	3072	
Q13972	RASGRF1	3060	
Q9BQ95	ECSIT	3054	
Q86Y56	HEATR2	3048	
P30533	LRPAP1	3026	
Q9NZQ3	NCKIPSD	3022	
Q96A33	CCDC47	3022	
Q96S59	RANBP9	2984	
P62380	TBPL1	2976	
O15068	MCF2L	2962	
P01241	GH1	2954	
Q9BXF6	RAB11FIP5	2944	
P43354	NR4A2	2922	
Q9H3Q1	CDC42EP4	2922	
Q10567	AP1B1	2906	
P48039	MTNR1A	2874	
O00499	BIN1	2862	
P62917	RPL8	2858	
Q9UHY1	NRBP1	2842	
P24390	KDELR1	2842	
NA	PIP3	2834	
O14653	GOSR2	2822	
O14640	DVL1	2806	
Q9Y4L1	HYOU1	2804	
Q9Y4D1	DAAM1	2792	
Q9UL25	RAB21	2788	yes
Q99541	PLIN2	2788	
Q96JA3	PLEKHA8	2788	
O60763	USO1	2778	
O75628	REM1	2752	yes
Q9Y5S2	CDC42BPB	2748	
Q9Y6W5	WASF2	2718	
P37108	SRP14	2700	
O75116	ROCK2	2680	
P61966	AP1S1	2676	
P04275	VWF	2662	
Q02241	KIF23	2650	
P48556	PSMD8	2648	
Q9UGI8	TES	2646	
P53609	PGGT1B	2636	
Q9Y3C7	MED31	2622	
Q99755	PIP5K1A	2556	
P35228	NOS2	2554	
P60866	RPS20	2550	
Q9C0E2	XPO4	2548	
Q9Y2D8	SSX2IP	2530	
Q9Y4G8	RAPGEF2	2528	
Q13576	IQGAP2	2512	
Q8WU20	FRS2	2462	
Q96KP1	EXOC2	2454	

P14672	SLC2A4	2446	
Q13905	RAPGEF1	2446	
Q9UPT6	MAPK8IP3	2444	
O15265	ATXN7	2436	
P48736	PIK3CG	2426	
Q0VDD7	C19orf57	2412	
O60603	TLR2	2400	
Q9GZT9	EGLN1	2396	
Q12802	AKAP13	2380	
O75489	NDUFS3	2374	
P04004	VTN	2368	
Q8NFH8	REPS2	2362	
O60318	MCM3AP	2346	
Q9NR96	TLR9	2332	
Q9H082	RAB33B	2322	yes
Q9NPF4	OSGEP	2306	
O43837	IDH3B	2302	
Q8IXJ6	SIRT2	2290	
Q9BQ39	DDX50	2274	
O94829	IPO13	2256	
Q9BV68	RNF126	2254	
P13804	ETFA	2246	
Q9Y5J5	PHLDA3	2234	
Q14451	GRB7	2200	
P23258	TUBG1	2196	
Q7L576	CYFIP1	2188	
Q9BUV8	C20orf24	2186	
Q5VYK3	ECM29	2182	
Q16512	PKN1	2174	
Q9Y3L3	SH3BP1	2170	
Q96D21	RASD2	2166	yes
O60383	GDF9	2124	
Q6UVK1	CSPG4	2122	
P08473	MME	2118	
Q5T5U3	ARHGAP21	2114	
Q92608	DOCK2	2106	
Q08945	SSRP1	2102	
Q9BRK5	SDF4	2096	
Q8NFF5	FLAD1	2088	
Q9UJM3	ERRFI1	2088	
Q7Z6B7	SRGAP1	2082	
P13861	PRKAR2A	2070	
O00458	IFRD1	2064	
Q9H0A6	RNF32	2052	
O60229	KALRN	2048	
P29372	MPG	2032	
Q9H4E5	RHOJ	2020	yes
Q96JB5	CDK5RAP3	2008	
O94941	UBOX5	2006	
Q9UHD8	SEPT9	2002	
P49789	FHIT	1980	
P57729	RAB38	1966	yes
Q99819	ARHGDIG	1964	

O00629	KPNA4	1956	
Q00341	HDLBP	1956	
Q7LDG7	RASGRP2	1954	
Q8TD19	NEK9	1944	
Q96D71	REPS1	1932	
P35125	USP6	1922	
Q13829	TNFAIP1	1920	
O00194	RAB27B	1920	yes
P78347	GTF2I	1920	
P02686	MBP	1914	
Q12772	SREBF2	1884	
Q12815	TROAP	1878	
P06454	PTMA	1874	
Q9UNN5	FAF1	1874	
Q16644	MAPKAPK3	1866	
Q96CN9	GCC1	1852	
P04632	CAPNS1	1844	
Q13064	MKRN3	1836	
Q9UNT1	RABL2B	1820	yes
O95347	SMC2	1816	
Q9NQU5	PAK6	1804	
Q9UK45	LSM7	1792	
P15170	GSPT1	1780	
Q68EM7	ARHGAP17	1774	
Q9BQ83	SLX1A	1758	
O60879	DIAPH2	1752	
P05060	CHGB	1752	
O43665	RGS10	1744	
Q8NF91	SYNE1	1728	
Q00722	PLCB2	1728	
P22460	KCNA5	1724	
P20823	HNF1A	1724	
Q14134	TRIM29	1682	
Q96BY6	DOCK10	1662	
Q5RL73	RBM48	1642	
P30154	PPP2R1B	1638	
Q9H1Y0	ATG5	1634	
Q9UNA4	POLI	1626	
Q13439	GOLGA4	1624	
Q13322	GRB10	1622	
Q15669	RHOH	1614	yes
P01375	TNF	1604	
Q8WUD1	RAB2B	1582	yes
O95373	IPO7	1582	
O60260	PARK2	1570	
P61158	ACTR3	1554	
O94925	GLS	1534	
O95819	MAP4K4	1532	
Q15257	PPP2R4	1504	
P31751	AKT2	1494	
Q9BYG4	PARD6G	1488	
Q7L804	RAB11FIP2	1476	
Q04637	EIF4G1	1474	

Q13148	TARDBP	1466	
Q96IZ7	RSRC1	1458	
Q9Y3E7	CHMP3	1450	
Q8N3R9	MPP5	1440	
Q15041	ARL6IP1	1438	
O75044	SRGAP2	1434	
Q9UIA9	XPO7	1426	
Q8N5V2	NGEF	1426	
P20290	BTF3	1412	
P52209	PGD	1408	
Q07890	SOS2	1398	
Q13535	ATR	1398	
P30556	AGTR1	1386	
Q9Y4I1	MYO5A	1384	
O60331	PIP5K1C	1364	
Q96QF0	RAB3IP	1348	
O43175	PHGDH	1348	
P30626	SRI	1346	
Q5JR01	EEF1A1	1338	
Q99828	CIB1	1338	
P18669	PGAM1	1322	
Q9H7P9	PLEKHG2	1318	
Q9BWH2	FUNDC2	1292	
O95714	HERC2	1286	
P57721	PCBP3	1270	
O75154	RAB11FIP3	1268	
Q9BST9	RTKN	1254	
O43559	FRS3	1246	
P05814	CSN2	1246	
P20309	CHRM3	1234	
P15531	NME1	1216	
O43747	AP1G1	1196	
O60684	KPNA6	1180	
P13591	NCAM1	1174	
Q9H4E7	DEF6	1160	
O60880	SH2D1A	1156	
O14974	PPP1R12A	1154	
P16035	TIMP2	1146	
Q14232	EIF2B1	1142	
P15822	HIVEP1	1126	
P02765	AHSG	1124	
Q8NHS7	PTPRS	1114	
Q6UB99	ANKRD11	1114	
P00747	PLG	1100	
Q9BPW5	RASL11B	1096	yes
Q4V328	GRIPAP1	1094	
Q8IZP1	TBC1D3	1082	
Q8NFW9	MYRIP	1078	
Q8WZA2	RAPGEF4	1066	
P48995	TRPC1	1062	
P67812	SEC11A	1058	
Q7L014	DDX46	1058	
P36404	ARL2	1054	yes

P10144	GZMB	1042	
Q5T0N5	FNBP1L	1042	
Q8IYU2	HACE1	1030	
Q14241	TCEB3	1024	
O43818	RRP9	1022	
P21359	NF1	1022	
P30622	CLIP1	1022	
P51955	NEK2	1000	
Q9H1K0	ZFYVE20	994	
Q8WUW1	BRK1	990	
O95835	LATS1	984	
P82094	TMF1	968	
Q86UR1	NOXA1	964	
Q9HCH5	SYTL2	942	
P04181	OAT	942	
Q9UM54	MYO6	930	
Q8WYP3	RIN2	926	
A1A4S6	ARHGAP10	906	
Q9Y2B9	PKIG	904	
Q9BY41	HDAC8	900	
Q9HD26	GOPC	900	
O95551	TDP2	890	
O00442	RTCA	888	
Q16625	OCLN	888	
Q14643	ITPR1	888	
O60890	OPHN1	876	
Q86VW2	ARHGEF25	876	
P53396	ACLY	870	
Q9UHV2	SERTAD1	860	
Q6FHU2	PGAM1	834	
Q9Y272	RASD1	818	yes
P30793	GCH1	794	
Q9NPH3	IL1RAP	780	
Q9BZY9	TRIM31	770	
Q96QZ7	MAGI1	758	
Q75N03	CBLL1	756	
P63162	SNRPN	742	
Q8WXI2	CNKSR2	710	
O14730	RIOK3	708	
Q9P0T4	ZNF581	708	
Q52LW3	ARHGAP29	700	
P15882	CHN1	694	
Q2M1Z3	ARHGAP31	694	
Q15637	SF1	686	
Q9BWA2	EWSR1	680	
Q9H4K1	RIBC2	672	
O95865	DDAH2	672	
Q9GZT6	CCDC90B	668	
O60477	DBC1	658	
P07197	NEFM	648	
O00305	CACNB4	648	
P26374	CHML	646	
P13497	BMP1	644	

P01127	PDGFB	638	
P21731	TBXA2R	630	
Q9HAW0	BRF2	624	
Q8N2S1	LTBP4	594	
Q5HYK7	SH3D19	592	
Q13017	ARHGAP5	590	
Q9UPM8	AP4E1	580	
O60784	TOM1	556	
Q5JSH3	WDR44	556	
P31153	MAT2A	556	
Q8N2Y8	RUSC2	554	
Q7Z465	BNIPL	548	
Q12829	RAB40B	546	yes
Q9P035	PTPLAD1	540	
Q969K3	RNF34	538	
P16452	EPB42	534	
P35555	FBN1	528	
Q96C24	SYTL4	522	
Q9BYV6	TRIM55	520	
Q8IW93	ARHGEF19	520	
B2RTY4	MYO9A	514	
P14314	PRKCSH	504	
Q8TEU7	RAPGEF6	500	
Q5T124	UBXN11	498	
Q01851	POU4F1	496	
Q3YEC7	RABL6	492	yes
Q9UH99	SUN2	486	
Q9HCK5	EIF2C4	484	
Q9NYN1	RASL12	474	yes
Q9BXR0	QTRT1	464	
O75832	PSMD10	460	
Q86YL6	RICS	458	
Q9NS91	RAD18	452	
O00522	KRIT1	448	
Q9Y4L5	RNF115	432	
P58062	SPINK7	426	
Q9Y4F9	FAM65B	424	
Q9NR80	ARHGEF4	418	
O43768	ENSA	416	
Q8N3F8	MICALL1	414	
O95267	RASGRP1	396	
Q9P286	PAK7	392	
Q12774	ARHGEF5	392	
Q15109	AGER	390	
O94827	PLEKHG5	388	
Q14088	RAB33A	386	yes
Q8IYJ3	SYTL1	384	
Q8TDW5	SYTL5	376	
Q8IZJ4	RGL4	374	
P50579	METAP2	370	
Q8IWN7	RP1L1	368	
Q9P0K1	ADAM22	364	
Q6DT37	CDC42BPG	356	

Q14449	GRB14	348	
P26367	PAX6	346	
Q9NP84	TNFRSF12A	344	
Q96G01	BICD1	336	
Q7L0Q8	RHOU	334	yes
P31629	HIVEP2	334	
P49848	TAF6	330	
P16389	KCNA2	328	
Q9H6R7	C2orf44	328	
Q9ULV0	MYO5B	326	
O15479	MAGEB2	326	
Q96HU1	SGSM3	324	
Q9NV70	EXOC1	310	
Q8TC07	TBC1D15	308	
O95704	APBB3	308	
P52757	CHN2	294	
P01009	SERPINA1	288	
Q9HA65	TBC1D17	286	
Q9P078		282	
Q9HC35	EML4	280	
Q5HYC2	KIAA2026	276	
C9J1V7	AAR2	276	
Q7Z628	NET1	274	
O00562	PITPNM1	262	
Q9HC57	WFDC1	262	
P28332	ADH6	262	
Q8TBN0	RAB3IL1	258	
P57078	RIPK4	254	
Q92738	USP6NL	254	
Q13459	MYO9B	252	
O75367	H2AFY	252	
P26885	FKBP2	250	
Q9UMR2	DDX19B	250	
O14787	TNPO2	246	
Q7Z698	SPRED2	246	
Q9Y600	CSAD	236	
Q53QZ3	ARHGAP15	236	
Q96NA2	RILP	234	
O95466	FMNL1	232	
P49366	DHPS	230	
Q13951	CBFB	228	
Q9BPX3	NCAPG	226	
Q12851	MAP4K2	226	
Q5JS13	RALGPS1	224	
Q00765	REEP5	222	
Q96QU8	XPO6	220	
O60645	EXOC3	218	
Q14820	SF1	216	
Q9H3F6	KCTD10	216	
Q8R2X8	Blzf1	212	
Q8N4G2	ARL14	212	yes
Q8NHH9	ATL2	204	
Q66PJ3	ARL6IP4	198	

Q9Y6X8	ZHX2	194	
Q6ZSZ5	ARHGEF18	192	
Q7Z444	ERAS	190	yes
P02775	PPBP	188	
Q9UKX7	NUP50	188	
Q8TDF6	RASGRP4	186	
Q8TCX5	RHPN1	184	
P00395	MT-CO1	180	
Q5VVX9	UBE2U	178	
P15622	ZNF250	176	
Q9NRR8	CDC42SE1	176	
Q9NRR3	CDC42SE2	176	
Q59HE9		176	
Q9Y689	ARL5A	174	yes
Q9NS15	LTBP3	174	
Q8NEU8	APPL2	170	
Q8N122	RPTOR	168	
O43324	EEF1E1	168	
Q9Y675	SNURF	166	
Q9NZL6	RGL1	166	
Q92565	RAPGEF5	164	
P16083	NQO2	162	
Q59EK9	RUNDC3A	160	
Q9P2R3	ANKFY1	158	
P22102	GART	154	
P51178	PLCD1	152	
Q96E17	RAB3C	148	yes
Q9NRD0	FBXO8	148	
O75843	AP1G2	148	
Q8TEC5	SH3RF2	142	
Q08289	CACNB2	140	
Q9UDV6	ZNF212	138	
B9EGS8	BRAP	138	
Q4VX76	SYTL3	136	
O95149	SNUPN	134	
P52824	DGKQ	134	
O95398	RAPGEF3	132	
Q8NEV8	EXPH5	132	
O15554	KCNN4	130	
O14559	ARHGAP33	130	
Q7LBR1	CHMP1B	128	
Q9H6Z4	RANBP3	126	
Q9UKK6	NXT1	126	
O75828	CBR3	126	
Q7RTN4	RTN3	120	
P07195	LDHB	118	
P03971	AMH	118	
P98174	FGD1	116	
Q8TBA6	GOLGA5	114	
O14763	TNFRSF10B	110	
Q9BYZ6	RHOBTB2	108	yes
Q92556	ELMO1	108	
P11712	CYP2C9	108	

Q6ZUM4	ARHGAP27	108	
Q9NR81	ARHGEF3	106	
Q9NTK1	DEPP	104	
P23368	ME2	104	
Q8N766	EMC1	104	
Q96F81	DISP1	102	
Q9H993	C6orf211	100	
Q92871	PMM1	92	
Q9UIL1	SCOC	92	
P48426	PIP4K2A	88	
Q6P3S6	FBXO42	88	
Q99932	SPAG8	82	
Q13368	MPP3	82	
Q6VN20	RANBP10	80	
Q92633	LPAR1	80	
Q8TD16	BICD2	80	
Q14644	RASA3	78	
Q99805	TM9SF2	76	
P58557	YBEY	74	
Q53G35		74	
Q5VT25	CDC42BPA	66	
Q99986	VRK1	64	
Q59ED0		64	
Q9UKI2	CDC42EP3	62	
O43889	CREB3	62	
O14613	CDC42EP2	62	
Q8K3H0	Appl1	62	
Q9H2G9	BLZF1	58	
P60468	SEC61B	56	
Q96HE3	EML4 protein	54	
Q9Y574	ASB4	54	
P85298	ARHGAP8	54	
Q8IV63	VRK3	48	
P08700	IL3	48	
O60831	PRAF2	46	
Q96S79	RASL10B	44	yes
Q9NWV8	BABAM1	44	
Q13427	PPIG	42	
Q96IZ5	RBM41	42	
O95235	KIF20A	40	
Q9ULL4	PLXNB3	38	
Q96IZ0	PAWR	38	
Q9Y312	AAR2	38	
Q14651	PLS1	38	
P50222	MEOX2	38	
P56470	LGALS4	38	
Q9Y330	ZBTB12	36	
Q13795	ARFRP1	36	yes
Q8WZ55	BSND	34	
Q7Z5R6	APBB1IP	34	
Q9Y4W6	AFG3L2	32	
Q86TW2	ADCK1	32	
P98171	ARHGAP4	30	

Q92805	GOLGA1	30	
Q8IWJ2	GCC2	30	
O95897	OLFM2	28	
Q96HD9	ACY3	26	
P59190	RAB15	24	yes
O75915	ARL6IP5	24	
Q15771	RAB30	22	yes
P01833	PIGR	22	
Q9HD47	RANGRF	22	
Q969Q5	RAB24	22	yes
Q7Z6U2	TBPL1	18	
P16989	CSDA	18	
Q3MII6	TBC1D25	16	
Q96DA2	RAB39B	14	yes
Q96LK0	CEP19	14	
O00478	BTN3A3	14	
Q6Q788	APOA5	12	
Q9P013	CWC15	10	
Q6XUX3	DSTYK	8	
Q9NR31	SAR1A	6	yes
O94844	RHOBTB1	4	yes
P59780	AP3S2	2	
O00488	ZNF593	0	
Q14588	ZNF234	0	
Q5T4F4	ZFYVE27	0	
Q9NRW7	VPS45	0	
B1GXI8	UNKL	0	
O14835	TXNL4A	0	
Q969M1	TOMM40L	0	
O14798	TNFRSF10C	0	
Q96A57	TMEM230	0	
O76062	TM7SF2	0	
O00445	SYT5	0	
O95907	SLC16A8	0	
Q9H446	RWDD1	0	
O75695	RP2	0	
Q99666	RGPD5	0	
Q969G6	RFK	0	
Q8IYK8	REM2	0	yes
Q7Z6M1	RABEPK	0	
O14966	RAB7L1	0	yes
P61018	RAB4B	0	yes
Q14964	RAB39A	0	yes
O95755	RAB36	0	yes
Q9ULZ3	PYCARD	0	
Q15673	PSD	0	
Q8IWE5	PLEKHM2	0	
O17874	O17874	0	
Q9Y5V0	ZNF706	0	
Q9Y473	ZNF175	0	
Q9Y3L5	RAP2C	0	yes
Q9Y2D4	EXOC6B	0	
Q9UPY8	MAPRE3	0	

Q9UP83	COG5	0	
Q9ULC3	RAB23	0	yes
Q9UKN1	MUC12	0	
Q9UHY7	ENOPH1	0	
Q9UG59	DKFZp564M2422	0	
Q9UBK7	RABL2A	0	yes
Q9P0J6	MRPL36	0	
Q9NX57	RAB20	0	yes
Q9NUZ0	IDH3B	0	
Q9NQG7	HPS4	0	
Q9NP90	RAB9B	0	yes
Q9HC74	SEPT9	0	
Q9HBH0	RHOF	0	yes
Q9HAM2	SH3RF1	0	
Q9H9P5	UNKL	0	
Q9H7X7	RABL5	0	yes
Q9H3U5	MFSD1	0	
Q96PY5	FMNL2	0	
Q96NB3	ZNF830	0	
Q96L33	RHOV	0	yes
Q96KC2	ARL5B	0	yes
Q96JJ3	ELMO2	0	
Q96HU8	DIRAS2	0	yes
Q96G28	CCDC104	0	
Q96CN4	EVI5L	0	
Q96BM9	ARL8A	0	yes
Q96AH8	RAB7B	0	yes
Q96A25	TMEM106A	0	
Q969V3	NCLN	0	
Q969Q4	ARL11	0	yes
Q92928	RAB1C	0	yes
Q92696	RABGGTA	0	
Q92544	TM9SF4	0	
Q8WXH6	RAB40A	0	yes
Q8TEJ3	SH3RF3	0	
Q8N972	ZNF709	0	
Q8N959	cDNA FLJ38330 fis, clor	0	
Q8N8L6	ARL10	0	yes
Q8N465	D2HGDH	0	
Q8N129	CNPY4	0	
Q8IZ81	ELMOD2	0	
Q86YS6	RAB43	0	yes
Q6X4W1	NSMF	0	
Q6PAL8	Dennd5a	0	
Q6IQ22	RAB12	0	yes
Q6IPX1	TBC1D3C	0	
Q6AI59	DKFZp781H0795	0	
Q60I27	ALS2CL	0	
Q5TBH8	GNPAT	0	
Q5HYI8	RABL3	0	yes
Q3SXY8	ARL13B	0	yes
Q3MIX3	ADCK5	0	
Q13637	RAB32	0	yes

Q0VGK0	Gabarapl1	0	
Q0P5N6	ARL16	0	yes
Q03386	Ralgds	0	
P56746	CLDN15	0	
P51157	RAB28	0	yes
A8MTZ0	BBIP1	0	
A6NIZ1	RP1BL	0	yes
A0S183	COX1	0	
A0N4V7	Tcr-alpha hCG	0	
O75052	NOS1AP	0	
Q8IVL1	NAV2	0	
P00414	MT-CO3	0	
Q13421	MSLN	0	
Q6ZW33	MICALCL	0	
Q7RTP6	MICAL3	0	
Q9Y664	KPTN	0	
Q14623	IHH	0	
Q9BW83	IFT27	0	yes
P15260	IFNGR1	0	
Q01629	IFITM2	0	
P08908	HTR1A	0	
O15228	GNPAT	0	
Q9BT04	FUZ	0	
Q96IH1	FSCN1	0	
P42685	FRK	0	
Q8NEG0	FAM71C	0	
O95661	DIRAS3	0	yes
O95057	DIRAS1	0	yes
P15169	CPN1	0	
Q96FZ7	CHMP6	0	
Q92496	CFHR4	0	
Q6NZY7	CDC42EP5	0	
Q6ZP82	CCDC141	0	
O60512	B4GALT3	0	
P21283	ATP6V1C1	0	
P27449	ATP6V0C	0	
Q8TDY4	ASAP3	0	
Q8N6S5	ARL6IP6	0	
P56559	ARL4C	0	yes
P40617	ARL4A	0	yes
Q17R89	ARHGAP44	0	
